# A Temperature-Monitoring Vaginal Ring for Measuring Adherence

**DOI:** 10.1371/journal.pone.0125682

**Published:** 2015-05-12

**Authors:** Peter Boyd, Delphine Desjardins, Sandeep Kumar, Susan M. Fetherston, Roger Le-Grand, Nathalie Dereuddre-Bosquet, Berglind Helgadóttir, Ásgeir Bjarnason, Manjula Narasimhan, R. Karl Malcolm

**Affiliations:** 1 School of Pharmacy, Queen’s University Belfast, Belfast, Northern Ireland, United Kingdom; 2 CEA, Division of Immuno-Virology, DSV/iMETI, IDMIT Center, Paris, France; 3 UMR-E1, Paris Sud University, Orsay, France; 4 Star-Oddi Ltd, Gardabaer, Iceland; 5 World Health Organization, Department of Reproductive Health and Research, Geneva, Switzerland; Centers for Disease Control and Prevention, UNITED STATES

## Abstract

**Background:**

Product adherence is a pivotal issue in the development of effective vaginal microbicides to reduce sexual transmission of HIV. To date, the six Phase III studies of vaginal gel products have relied primarily on self-reporting of adherence. Accurate and reliable methods for monitoring user adherence to microbicide-releasing vaginal rings have yet to be established.

**Methods:**

A silicone elastomer vaginal ring prototype containing an embedded, miniature temperature logger has been developed and tested *in vitro* and in cynomolgus macaques for its potential to continuously monitor environmental temperature and accurately determine episodes of ring insertion and removal.

**Results:**

*In vitro* studies demonstrated that DST nano-T temperature loggers encapsulated in medical grade silicone elastomer were able to accurately and continuously measure environmental temperature. The devices responded quickly to temperature changes despite being embedded in different thickness of silicone elastomer. Prototype vaginal rings measured higher temperatures compared with a subcutaneously implanted device, showed high sensitivity to diurnal fluctuations in vaginal temperature, and accurately detected periods of ring removal when tested in macaques.

**Conclusions:**

Vaginal rings containing embedded temperature loggers may be useful in the assessment of product adherence in late-stage clinical trials.

## Introduction

It has long been assumed that use of sustained or controlled release delivery systems for vaginal administration of microbicides to prevent infection with human immunodeficiency virus (HIV) will lead to increased microbicide product adherence, acceptability and efficacy compared with more conventional, coitally-dependent, vaginal formulations [1–4]. Indeed, based on adherence data from other clinical indications [5–7], including hormonal contraception for which long-acting depot injections, sub-dermal implants, transdermal patches and vaginal rings are available [8,9], the case for sustained/controlled release of HIV microbicides is generally well made and widely accepted. The first convincing evidence in support of the vaginal microbicide concept came from the CAPRISA 004 trial in which a tenofovir gel reduced HIV acquisition by an estimated 39% [10–12]. However, adherence estimates based on vaginal applicator returns indicated that HIV incidence was 54, 38 and 28% lower in the tenofovir gel arm for high, intermediate and low adherers, respectively, demonstrating unequivocally that high adherence is key to microbicide effectiveness.

One of the major challenges for the HIV microbicide field is the accurate (and preferably quantitative) measurement of adherence in late stage clinical trials [3,13,14]. Generally, methods for measuring adherence can be divided into two distinct categories. Direct measures of adherence, also referred to as “biomarkers”, are substances or effects whose presence or absence indicates that a biological or pharmacological process has occurred in response to a drug [14]. Indirect measures of adherence comprise two major sub-categories: “objective measures” and “self-report measures”, both reliant on the observations or reports of clinicians, trial participants, or others [14]. Some of the methods reported for assessing adherence are specific to a particular product type. For example, several advanced vaginal gel applicators have been developed, either containing a dye that changes colour upon exposure to mucin or that record the date and time that the piston is depressed into the applicator barrel [15]. Human core body temperature normally fluctuates between 36.1°C and 37.8°C depending on the individual and fluctuates by about 0.5°C over a 24 h period, with a minimum in the early hours of the morning and a maximum in late afternoon [16]. Environmental temperatures in many parts of the world also follow a diurnal cycle, but significantly the temperature range is much more than 0.5°C over 24 hours. For example, Harare, Zimbabwe in sub-Saharan Africa had an average diurnal air temperature range in August 2013 from 10 to 28°C over 24 h. In November this range was 16 to 34°C [17]. Considered to be proportional to core body temperature as in the case for rectal temperature measurement, the recording of vaginal temperature then offers an alternative and interesting biomarker option for monitoring adherence to microbicide-releasing vaginal rings. Recorded temperature deviations away from the limited diurnal range observed within the vaginal vault of a patient could be used as indicative evidence that a ring device has been removed and left exposed to the much more varied environmental temperature range.

Controlled drug release vaginal rings are already available for hormonal contraception (Nuvaring) and estrogen replacement therapy (Estring and Femring). For each of these marketed devices, the drug compound(s) is incorporated into a non-biodegradable polymeric reservoir that is then overmolded or encapsulated with a non-medicated polymeric sheath. The sheath layer controls precisely the rate of drug delivery to the vagina. Vaginal rings offer certain advantages over more conventional semi-solid and tablet formulations for vaginal delivery of drugs, most notably (i) long term use over weeks or months, (ii) controlled drug administration, and (iii) potentially increased user adherence. For these reasons, there is considerable interest in developing vaginal rings for administration of antiretroviral agents as a strategy to reduce or prevent the sexual transmission of HIV. A dapivirine-releasing vaginal ring is presently being evaluated in two Phase III clinical studies in Africa. In addition to determining the 25 mg dapivirine ring’s safety and effectiveness, the MTN-020—ASPIRE study has enrolled up to 3,476 HIV-negative women across 15 clinical research sites in Africa over 2 years, and is also designed to assess women’s adherence to the ring, using various interview methods, measurement of dapivirine concentrations in blood and vaginal fluid, and determination of residual dapivirine quantities in used rings.

**Fig 1:**
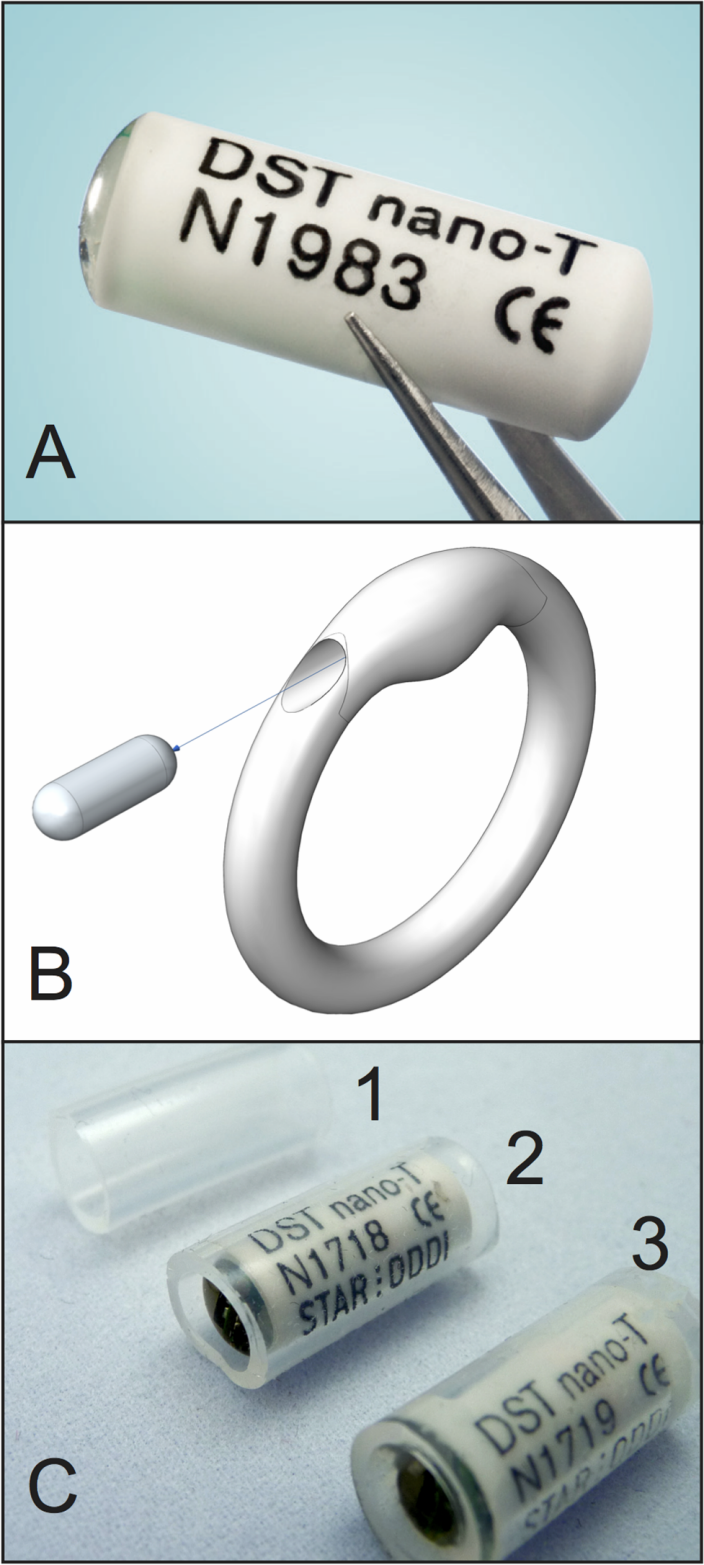
A—DST nano-T temperature logging device, as supplied; B—illustration of vaginal ring device with cavity for insertion of temperature logger. C—construction of temperature logging device encapsulated in silicone elastomer tubing for vaginal testing in macaques; C1 shows the silicone elastomer tubing (8.0 mm overall diameter); C2 shows the logger inserted into the silicone elastomer tubing; C3 shows the tubing end-sealed with silicone elastomer.

The relatively large size of vaginal rings (for example those manufactured from silicone elastomer or ethylene vinyl acetate (EVA)) compared with more conventional vaginal dosage forms, their method of manufacture, and their non-degradable properties *in vivo* offer the possibility of additionally incorporating a miniature temperature-recording device into the ring body. Assuming (i) the embedded temperature-recording device could accurately and periodically record environmental temperature, (ii) internal vaginal temperature is significantly different from external ambient temperature, and (iii) that the vaginal ring is returned to the clinic after use, it should be possible to use such a ring to accurately monitor user adherence. Ideally, the temperature-recording device would not adversely affect the drug delivery function. A similar approach using a plastic vaginal probe has previously been reported for the continuous monitoring of vaginal temperature in cattle [18]. Also, within the last year, the Ovularing temperature-monitoring vaginal ring system (VivoSensMedical GmbH, Leipzig, Germany), comprising a temperature sensor embedded in a thermoplastic ring body and a readout kit, has been marketed as a diagnostic tool for fertility and natural family planning. Here, we report proof-of-concept of a novel, vaginal, temperature-recording device comprising a miniature temperature recording implant (Fig 1A) encapsulated within a non-medicated silicone elastomer vaginal ring (Fig 1B). This silicone elastomer ring has dimensions similar to both the marketed drug-releasing vaginal rings Estring and Femring and to the current dapivirine-releasing ring.

## Materials and Methods

### Materials

DST nano-T (6.0 mm diameter, 17.0 mm length, mass 1 g) and DST micro-T (8.3 mm diameter, 25.4 mm length, mass 3.3 g) implantable temperature loggers were supplied by Star-Oddi (Gardabaer, Iceland). The loggers are housed in a waterproof, biocompatible epoxy/alumina case, are individually calibrated with a resolution temperature of 0.032°C, an accuracy of ± 0.2°C (guaranteed for one year) and a memory capacity of 5,248 and 43,476 measurements, respectively. SL51T button temperature data loggers were purchased from Signatrol (Gloucester, UK); these are housed in a cylindrical stainless steel body (17.0 mm diameter, 6.0 mm height) and offer a temperature resolution of 0.5°C, accuracy of ± 1.0°C, and a capacity for 43,477 measurements. A two-part, addition cure, medical grade, translucent, liquid silicone elastomer kit (DDU-4320) with final durometer hardness of 20 ShoreA was purchased from NuSil Technology (Carpinteria, CA, USA). Translucent, addition-cured, silicone elastomer tubing having 6.0 mm internal diameter, various wall thicknesses (1.0, 1.5, 2.0 and 3.0 mm) and Shore A hardness of 60 was purchased from Polymax (Hampshire, UK). Simulated vaginal fluid (SVF) was prepared using analytical grade reagents according to a method described previously [19].

### Manufacture of temperature loggers encapsulated in silicone elastomer

DST nano-T loggers were first configured to synchronously record temperature at sampling intervals of either 8 or 60 min. Each logger was inserted into a 20.0 mm length of silicone elastomer tubing (various wall thicknesses), leaving 1.5 mm clearance at each end. Part A (1.0g) and Part B (1.0g) of DDU-4320 silicone elastomer were mixed using a SpeedMixer DAC 150 FVZ-K (Hauschild, Germany) operating at 3000 rpm for 60 s. The tubing with nano-T inserted was then positioned vertically and the pre-mixed DDU-4320 material was poured into the open end and then cured for 120 s using a manual heat gun set at 600°C. The same procedure was repeated at the other end of the tube to produce a fully sealed device (Fig 1C).

### Effect of temperature recording interval and temperature change on the *in vitro* response of temperature monitoring devices tested over 8 h period

DST nano-T devices as supplied (non-encapsulated), encapsulated in silicone tubing of wall thickness 1.0 mm, or encapsulated in silicone tubing and immersed in 10 mL pre-heated SVF were placed in an orbital shaking incubator (Infors HT Unitron, Switzerland, 37^°^C, 60 rpm, 25 mm orbital throw) and the temperature monitored over 8 h. SL51T loggers configured to record temperature at 1 min intervals were also placed in the shaking incubator and in the laboratory to record background temperatures over the same 8 h period. The DST nano-T devices were removed from the incubator at 120 min, returned at 240 min, and then finally removed at 360 min. After each removal, the devices were placed on a laboratory bench, close to a SL51T logger device recording ambient temperature. Each experiment was repeated three times.

### Effect of silicone elastomer tubing wall thickness on the *in vitro* response of temperature monitoring devices

The effect of silicone elastomer sheath thickness (0, 1.0, 1.5, 2.0 and 3.0 mm) on the *in vitro* temperature responses of DST nano-T devices encapsulated in silicone elastomer (Fig 1C) and incubated in SVF heated to 37°C, were assessed over a 12 h period using a temperature-recording interval of 20 sec. At scheduled times during the 12 h testing period, the devices were removed from the incubated SVF for 20, 30 or 40 min and placed on the laboratory bench (average 20°C), after which they were once again returned to the SVF. The 20 min removal interval was selected as this was known from previous experiments (unpublished) to be sufficient for unsheathed loggers to cool down to laboratory temperatures from the 37°C incubated SVF. This interval was increased to a maximum of 40 min in case of delayed temperature recording response due to the effects of varying the sheath thickness.

### Effect of different insertion/removal schedules on the *in vitro* response of temperature monitoring devices tested over a 7-day period

DST nano-T devices, encapsulated in 1.0 mm thick silicone tubing, immersed in 10 mL SVF within an orbital shaking incubator and configured to record temperature every 8 min, were tested for temperature response using insertion/removal schedules that simulated different vaginal ring *in vivo* use scenarios. In the first scenario, the device was placed in the simulated vaginal environment for 7 days continuously, reflecting a ring user being fully compliant. In the second scenario, the device was placed in SVF for 4 days followed by removal for the remaining 3 days of the study, simulating a partially compliant user. In the third scenario, an attempt was made to assess how the data might be interpreated from a user who has a random pattern of compliance using the ring. The device was periodically inserted and removed over a 12 h period according to a schedule determined by the researcher, and to which the study coordinator was blinded. In the fourth scenario, we assessed the sensitivity of the device to removal duration, by periodically removing the device from SVF for progressively longer time periods (5, 10, 15, 20, 30, 45 and 60 min) during each hour of the study period.

### Monitoring of vaginal temperature in macaques

The clinical potential of a temperature-recording vaginal ring for monitoring product adherence was tested in female cynomolgus macaques (weight range 3–5 kg; n = 3). Macaques were selected to trial the device as they are commonly used for preclinical testing of vaginal HIV microbicide candidates and they also exhibit similar basal body temperatures and circadian rhythms to women. For each animal, a DST micro-T device was implanted subcutaneously under anaesthesia in the upper back, between scapulas to avoid scratches, on day 0 and remained in place for 32 days. A silicone elastomer encapsulated DST nano-T device (1.0 mm wall thickness, 8 min temperature recording interval) was inserted atraumatically and under anaesthesia into the bottom of the vaginal vault of each animal on day 25. Animal 1 retained the vaginal device until the end of the study on day 32, designed to simulate a patient demonstrating complete compliance using the ring device continuously. Animal 2 retained the vaginal device until day 28, when it was subsequently removed and stored in the laboratory environment until the end of the study. This scenario was to mimic a patient who would utilise the ring compliantly for a period of time but then remove it for the remainder of the trial period. For animal 3, the vaginal device was periodically removed and inserted; on days 26, 27 and 29, the device was removed for 30 min, 19 h and 30 min, respectively, before final removal on day 32 as per the other animals. The purpose of this removal schedule was to simulate a scenario where a patient might remove their ring repeatedly and randomly during a trial, the duration of the short removal periods being dictated by the sedation protocol used. Following removal from the macaque vagina, encapsulated DST nano-T devices were immediately washed with PBS buffer and placed into a sealed 50 mL Falcon tube until reinsertion or the end of the study.

Adult sexually mature female cynomolgus macaques (Macaca fascicularis) imported from Mauritius were housed in the facilities of the Infectious Disease Models and Innovative Therapies (IDMIT) center, part of the “Commissariat à l’Energie Atomique et aux Energies Alternatives” (CEA, Fontenay-aux-Roses, France). Non-human primates (NHP) are used at the CEA in accordance with French national regulations and under the supervision of national veterinary inspectors (CEA Permit Number A 92-032-02). The CEA complies with the Standards for Human Care and Use of Laboratory Animals, of the Office for Laboratory Animal Welfare (OLAW, USA) under OLAW Assurance number #A5826-01. The use of NHP at the CEA is in conformity with the recommendations of European Directive (2010/63, recommendation N°9). The animals were used under the supervision of the veterinarians in charge of the animal facility. This study was approved and accredited under statement number A14-036 by the ethics committee “Comité d'Ethique en Expérimentation Animale du CEA” registered under number 44 by the French Ministry of Research. Animals were housed in adjoining individual cages allowing social interactions, under controlled conditions of humidity, temperature and light (12-hour light/12-hour dark cycles). Water was available ad libitum. Animals were monitored and fed 1–2 times daily with commercial monkey chow and fruits by trained personnel. Macaques were provided with environmental enrichment including toys, novel foodstuffs and music under the supervision of the CEA Animal Welfare Body. Experimental procedures (animal handling, vaginal ring insertions and removal, blood samplings) were conducted after animal sedation with ketamine hydrochloride (Rhône-Mérieux, Lyon, France, 10 mg/kg). No animal was sacrificed for this study.

### Assessment of sampling interval on DST nano-T battery lifetime

Battery life as a function of sampling interval was measured using a current consumption method. The current during the measurement preparation, measurement phase and memory writing was measured through a 220Ω resistor and an oscilloscope Tektronix TDS2024. The sleep current was measured directly through the battery with a Keithley 6485 picoammeter. The measurements where used to calculate the battery life for various sampling intervals ranging from 6 seconds to 60 minutes.

## Results

### *In vitro* temperature-monitoring experiments

**Fig 2:**
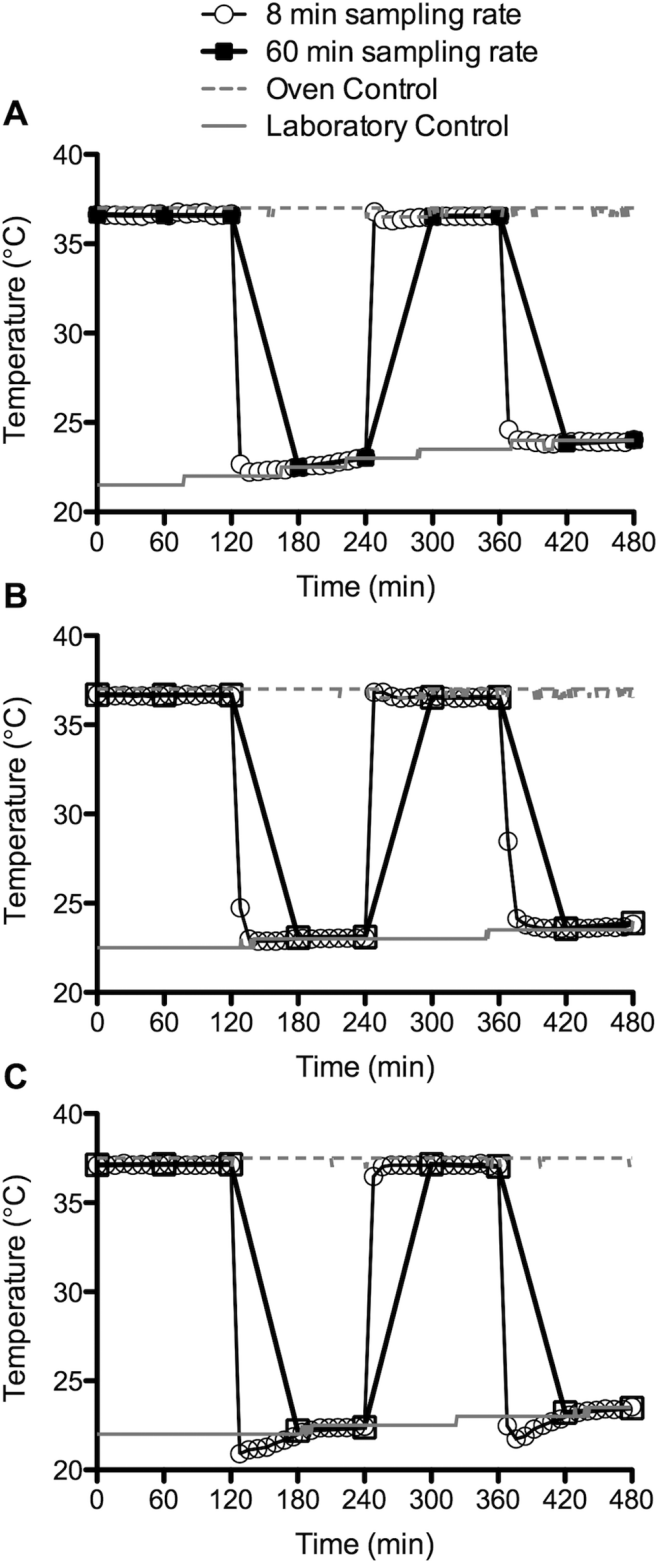
*In vitro* testing of DST nano-T temperature logging devices using sampling rates of 8 and 60 min. Devices were placed in a shaking orbital incubator set to 37°C, removed from the incubator at 120 min, returned to the incubator at 240 min, and then finally removed at 360 min. A—devices as supplied, i.e. non-encapsulated. B—devices sealed in silicone elastomer tubing. C—devices sealed in silicone elastomer tubing and placed in 10 mL of simulated vaginal fluid contained in a glass beaker.

The temperature data recorded by the non-encapsulated DST nano-T devices placed directly into the controlled temperature incubator are presented in Fig 2A. Close to 120 min, the profile for the device set to measure temperature every 8 min showed sequential decreases in temperature from 36.67°C to 22.68°C and then finally to 22.23°C, corresponding with removal of the device from the incubator and return to baseline ambient laboratory temperature. A similar temperature decrease was observed for the device recording temperature every 60 min (from 36.60 to 22.48°C), although the longer sampling interval means that the exact time of removal cannot be determined to the same degree of accuracy. Following reinsertion of the devices into the incubator, the device set to sample every 8 min was able to define the insertion time as between 240 and 248 min, compared to the 60 min window (240–300 min) defined by the device with the longer sampling interval. The second removal timepoint at 360 min showed entirely similar patterns. The laboratory and incubator control temperatures, monitored by the SL51T devices, closely matched the upper and lower temperatures measured by the DST devices (Fig 2A).

Fig 2B shows the temperature data collected when the same removal/insertion schedule and protocol was used with DST nano-T devices fully encased in a silicone elastomer sheath of 1.0 mm thickness. From an experimental perspective, we were interested to learn whether encapsulation with silicone elastomer would modify the temperature response of the DST device; the thermal conductivity of silicone rubber is about 0.2 W.m^-1^.K^-1^, some 10-fold greater than the value for air (0.024 W.m^-1^.K^-1^) [20]. Despite the difference in thermal conductivity, a temperature response profile very similar to the non-encapsulated device was observed. The only points of difference observed were the increased time intervals for the devices to cool from 37°C to laboratory temperature, as evidenced by the additional data points at 128 min (24.74°C) and 368 min (28.47°C) in the two cooling portions of the profile. By comparison, a delayed temperature response was not observed in the heating cycle following re-insertion into the incubator at 240 min.

More significant differences in temperature response were observed when the silicone elastomer encapsulated devices were placed in pre-warmed SVF within the incubator. Here, the SVF medium was intended to model the vaginal environment. Under these conditions, the devices displayed the most rapid temperature decrease, with the maximum and minimum temperature values being recorded unequivocally within a single 8 or 60 min sampling interval (Fig 2C). This is consistent with the high thermal conductivity value for water (0.58 W.m^-1^.K^-1^). More significantly, following removal of the 8 min sampling device at 120 min, the device then recorded a minimum temperature of 20.2°C at the next sampling timepoint (128 min); this recorded temperature was significantly lower, by approximately 1°C, than the laboratory temperature control. A similar decrease to below ambient temperature was also observed the second time the device was removed from the incubator at 360 min. These sub-ambient temperatures are attributed to an endothermic evaporative cooling process that occurs as the water component of SVF evaporates from the surface of the silicone sheath during the removal period.

**Fig 3:**
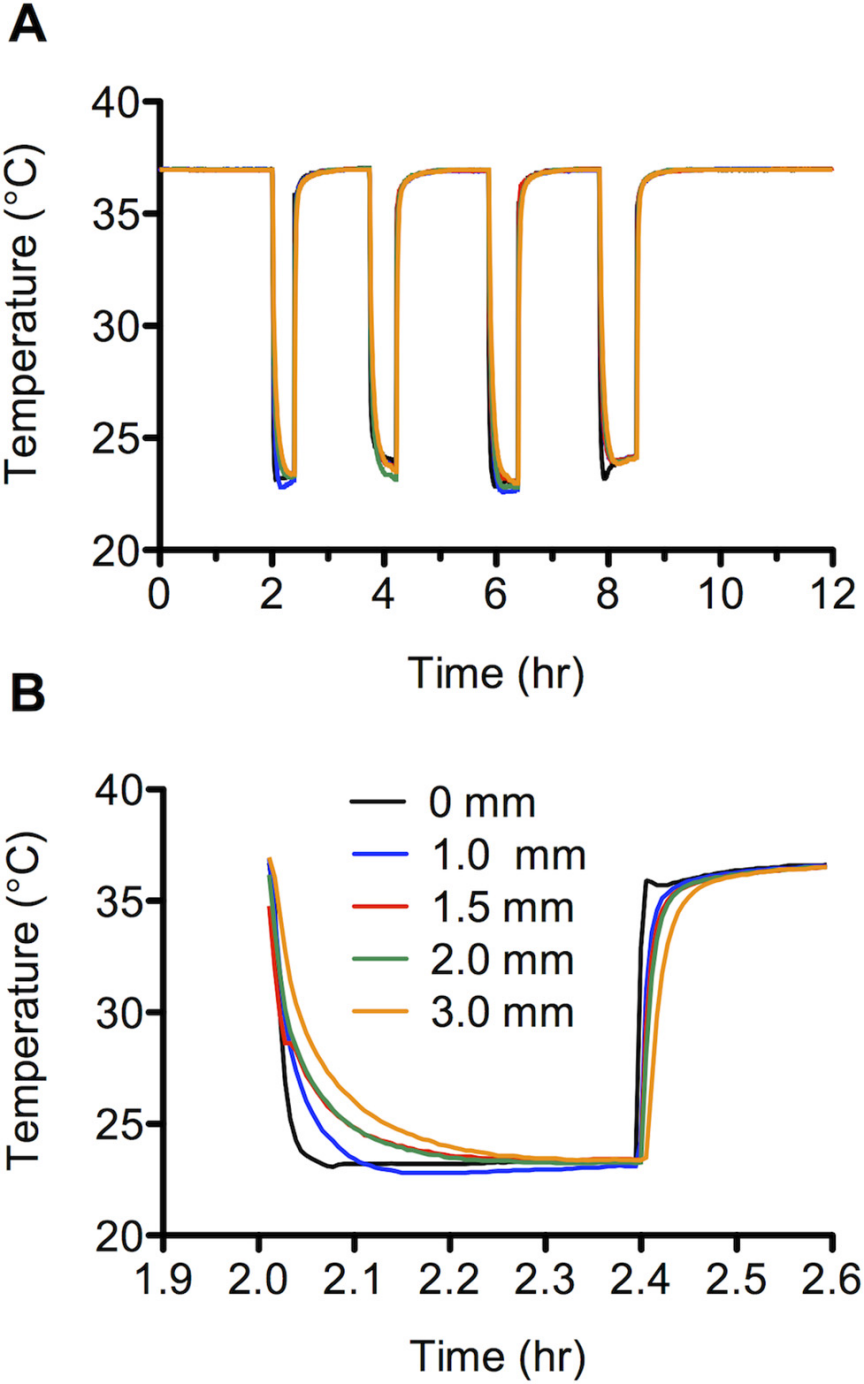
Effect of silicone elastomer sheath thickness on the *in vitro* temperature responsiveness of encapsulated DST nano-T devices in SVF. A—Four removals and re-insertions were performed. The removal periods were 20, 30, 30 and 40 min. Temperature was recorded at 20 sec intervals; data points are not shown for sake of clarity. B—Magnified view of the first removal and re-insertion period, showing the cooling and heating trends as a function of sheath thickness.

The effect of silicone elastomer wall thickness on the *in vitro* temperature responsiveness of encapsulated DST nano-T devices in SVF is presented in Fig 3. A general trend of delayed temperature responsiveness with increasing wall thickness was observed immediately following device removal and insertion (Fig 3B). This effect is associated with the specific heat capacity of the silicone rubber around the logger device and whilst a specific capacity for our silicone system is unavailable, a typical value is 0.8 J/g°C. Taking the example of the 1.0 mm wall thickness sheath (mass = 1.10 g), to decrease the temperature of the sheath from 37°C to 25°C would require the loss of approximately 10 J of heat energy. So there is a delay in the temperature response as the sheath loses this thermal energy store, and conversely when reinserted, the sheath absorbs this energy and delays the recording of the increase in temperature. These effects will increase proportionally with sheath wall thickness as the mass of silicone around the logger increases, so for clinical applications it would be preferable to have a wall thickness as thin as possible around the logger.

**Fig 4:**
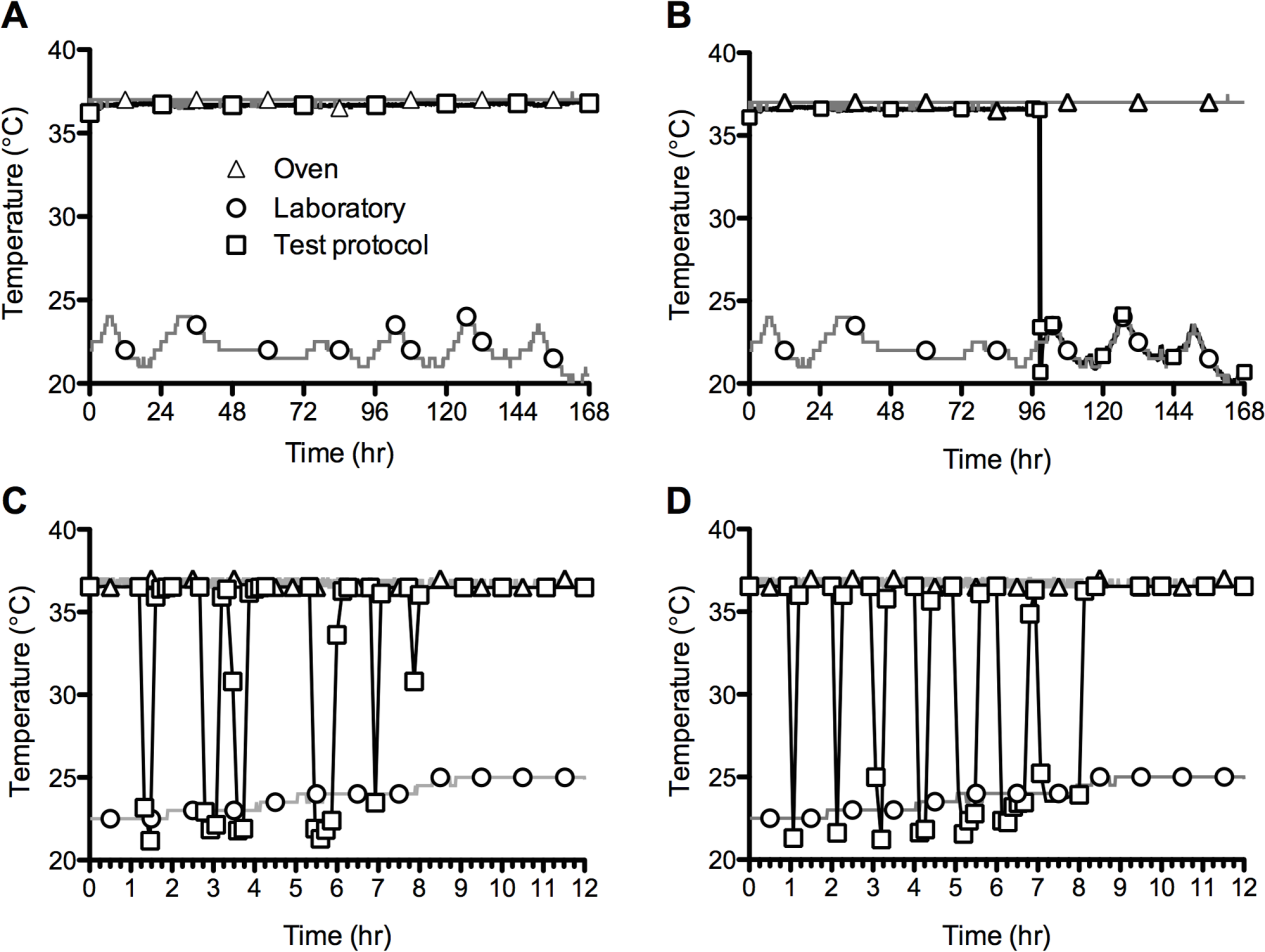
*In vitro* testing of silicone elastomer encapsulated DST nano-T devices immersed in incubated SVF, showing the temperature responses subject to four different use schedules. In each case, the sampling interval was 8 min. A—device maintained at 37°C; B—device removed from incubator at 96 h; C—device removed and returned to incubator numerous times during a 12 h period; D—device removed from incubator for progressively longer time periods before being returned.

Fig 4A shows the temperature vs. time profile recorded for an encapsulated device immersed continuously in SVF within a 37°C incubator for 7 days. No significant differences in temperature were observed compared to the control incubator temperature. Laboratory temperature measurements ranged between 20.0 and 24.0°C, peaking each day during Monday to Friday when central heating was available in the laboratory and showing no daily peak at the weekend when central heating was switched off. In Fig 4B, the device was removed from the SVF after 4 days; the removal time is precisely defined as occurring between 98 h 32 min and 98 h 40 min, within a single 8 min time interval. Further evidence for evaporative cooling was also observed, with the device recording a sub-ambient minimum temperature of 20.69°C at 98 h 48 min. Fig 4C shows the 12 h temperature vs. time profile for a device exposed to a more demanding removal and insertion schedule, and where the study coordinator was blinded to the schedule devised by the laboratory researcher. This experiment aimed to more closely simulate the blinded perspective of a clinical trial coordinator. The profile clearly shows six device removal and re-insertion episodes occurring within the first eight hours. A comparison of the temperature data recorded by the device and the removal/insertion times accurately recorded by the laboratory researcher demonstrated that removal times were accurately predicted to within ± 3 min. Given that the devices were configured to record temperature values every 8 min, the error could have as high ± 8 min. Based on the recorded data, it was also possible to predict the total period of removal of the device to within ± 8 min. Finally, Fig 4D shows the temperatures profiles for devices removed for progressively longer time intervals (5–60 min). In each case, the removal interval is clearly resolved by the recorded temperature data. Once again, Fig 4C and 4D show the sub-ambient cooling effect attributed to evaporation of water from the devices during the removal period. A summary of all in-vitro temperature range data is found in Table 1.

**Table 1:** Statistical data for DST nano-T devices placed in various *in vitro* and *in vivo* environments.

	Mean temperature ± SD (°C)	Min. temperature (°C)	Max. temperature (°C)	Range (°C)
Incubator, non-encapsulated	36.58 ± 0.12	36.30	36.80	0.50
Incubator, encapsulated	36.62 ± 0.09	36.49	36.83	0.34
Incubator, encapsulated, in SVF	37.12 ± 0.03	37.03	37.20	0.17
Macaque, vagina ^#^	36.44 ± 0.84	34.71	38.17	3.46
Macaque, subcutaneous ^#^	35.37 ± 0.89	33.15	37.45	4.30
Macaque, laboratory control ^#^	22.0 ± 0.1	21.7	22.3	0.6

^#^ Data obtained from Fig 6A.

8 min sampling rate was used for all devices, except the macaque laboratory control where temperature was recorded every 30 min.

### Macaque studies

**Fig 5:**
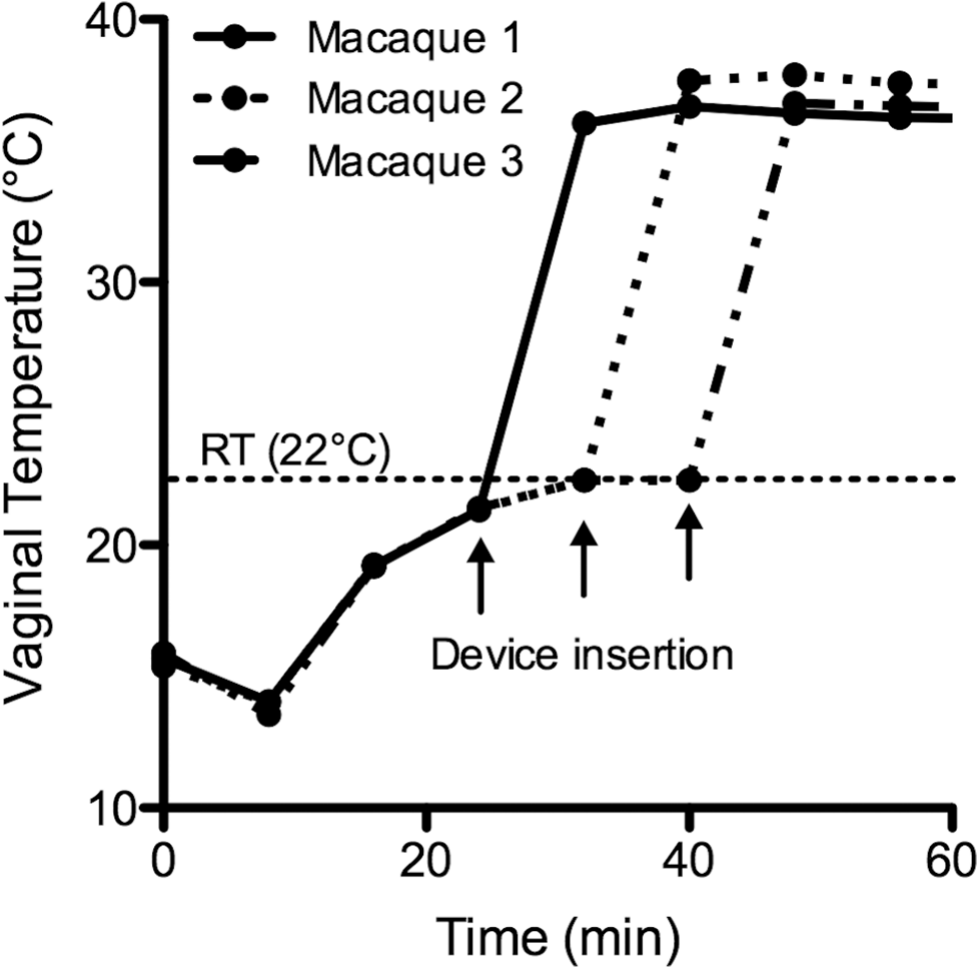
Temperature response of the silicone elastomer-encapsulated temperature logging devices before, during and immediately after vaginal insertion in cynomolgus macaques (n = 3). The arrows indicate vaginal insertion of the device. The dashed line (RT) indicates ambient laboratory temperature.

The sequential timings for vaginal insertion of the encapsulated devices in the three macaques are clearly captured in the temperature vs. time profiles presented in Fig 5. For all animals, the change from room temperature to body temperature occurred within a single 8 min sampling interval. Changes in ambient environmental temperature associated with initial transfer of the devices into the macaque facility are also clearly observed between 0 and 40 min, during which the recorded temperature increases from ~15 to 22°C.

**Fig 6:**
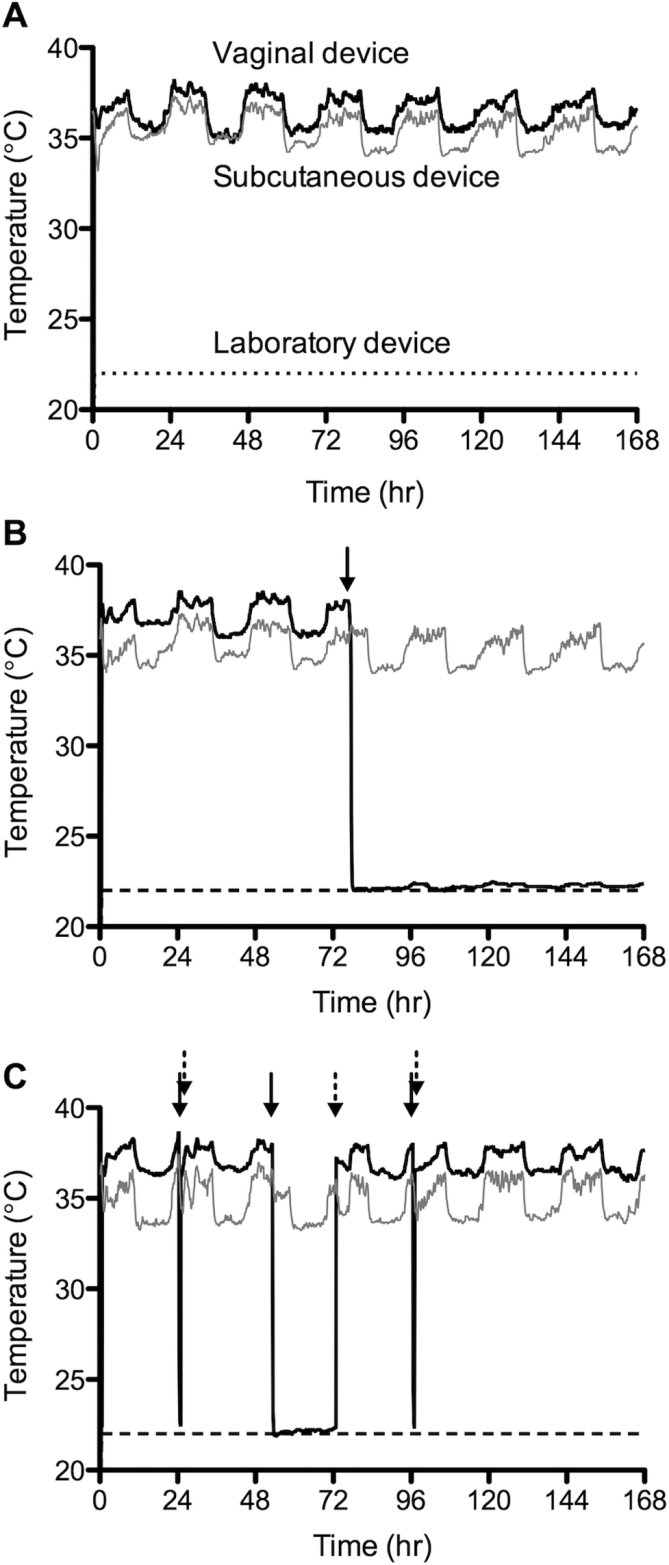
Temperature response versus time for vaginally and subcutaneously administered temperature loggers in cynomolgus macaques. Each graph shows a representative plot for a single macaque. Solid and dashed arrows indicate removal and re-insertion of the vaginal device, respectively. Laboratory temperature as measured by a control temperature logging device is indicated by the dashed line at ~22°C. A—vaginal device worn continuously over a 7-day period. B—vaginal device removed after 3 days placement. C—vaginal device removed and reinserted on three different occasions during the 7-day period.

**Fig 7:**
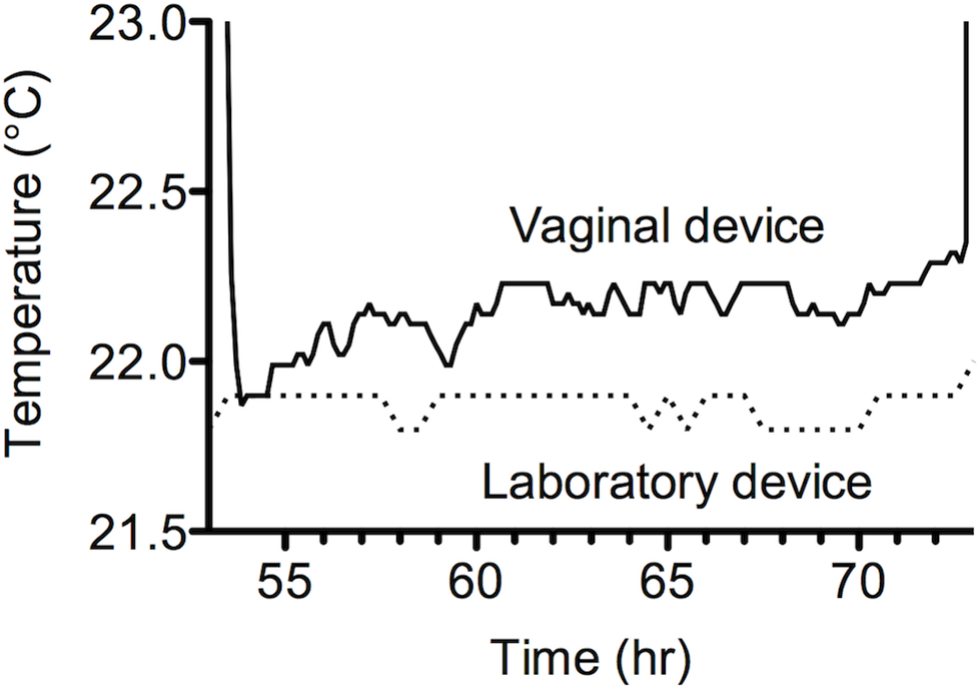
Evaporative cooling effect following removal of the silicone elastomer encapsulated DST nano-T device from the macaque vagina. Temperatures recorded by the vaginal device drop immediately after removal and increase with time. The lower temperatures measured soon after removal are due to evaporation of PBS wash buffer from the encapsulated device. As the water evaporates, the measured temperature rises. To more fully appreciate the evaporative cooling phenomenon, the baseline laboratory data, which is relatively low due to remote location of the sensor, can be shifted to 22.3°C.

The temperature data recorded during 7-day continuous use of subcutaneous and vaginal devices are in relative agreement with each other (Fig 6A). Both profiles clearly show a diurnal pattern comprising higher temperatures during daytime activity and lower temperatures during night-time inactivity, this showing very good agreement with the diurnal cycle observed in a woman’s basal body temperature. However, the vaginal temperatures are consistently higher than those measured subcutaneously, reflecting the fact that internal vaginal temperatures are more representative of core body temperature that those measured close to the external skin surface [21]. The temperature profile following removal of the device from the macaque vagina is presented in Fig 6B, and clearly defines the removal timepoint (77 h 44 min following initiation of temperature recording; or 2:44 pm on day 28 of the study). This correlates very closely with the study log diary in which removal of the device from this macaque is recorded at 14:45 on day 28. Fig 6C shows tee separate occasions when the vaginal device was removed and re-inserted in the macaque. Once again, the data shows excellent agreement with the times recorded in the values entered into the study log diary by the laboratory veterinarian. Closer inspection of the second vaginal device removal period at 53 hr (Fig 7) illustrates that the evaporative cooling effect previously observed *in vitro* was also observed in the macaques, albeit this time attributed to evaporation of the PBS wash buffer rather than SVF. Between 52.36 and 71.68 hr, during which the device is removed, the device temperature rises from 21.87 to 22.35°C. This compares with a laboratory temperature of 22.0–22.3°C recorded during this period. Blood sampling of the 3 animals revealed no significant changes in their haematology. No toxicological analysis was performed on the animals but veterinary staff did not observe any signs of vaginal irritation during the study.

### Influence of sampling interval on battery life

**Fig 8:**
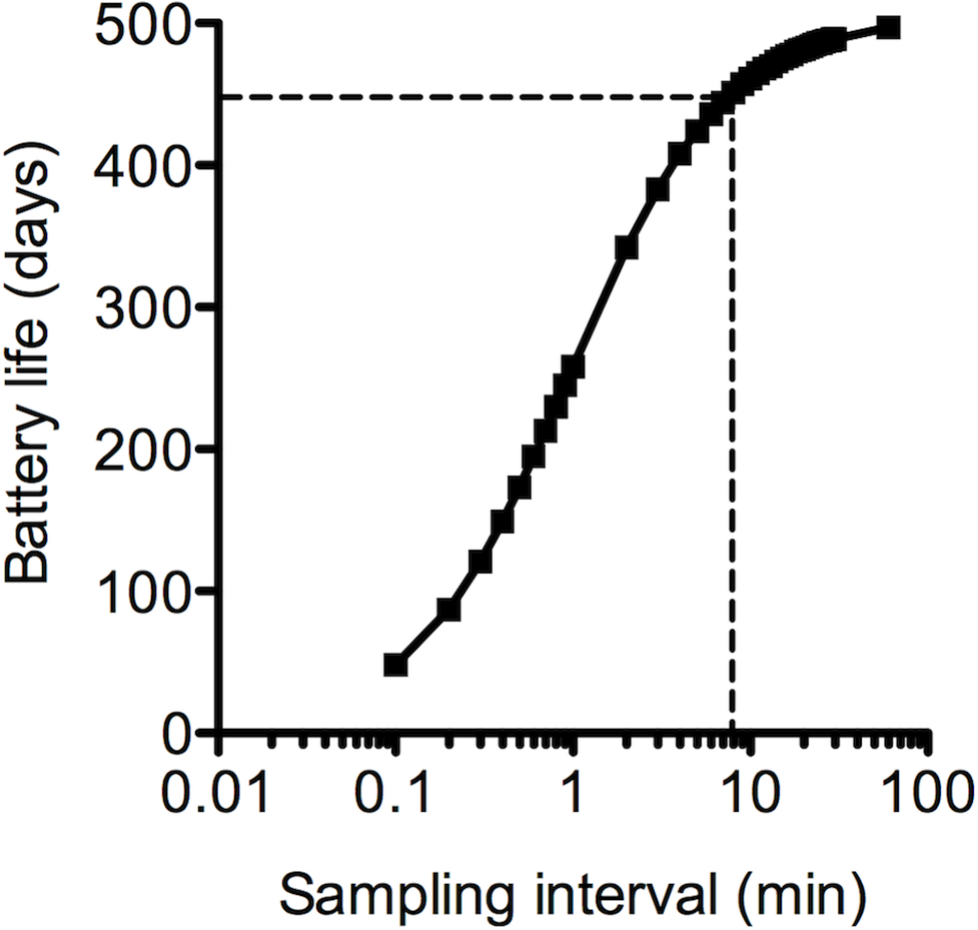
Effect of DST nano-T temperature sampling interval on measured battery life. Dotted line indicates battery life at 8 min sampling interval.

DST nano-T battery life was significantly influenced by the temperature-sampling interval, ranging from approximately 50 days with a 6 sec interval to 500 days with a 100 min interval (Fig 8). For the 8 min sampling interval used in the macaque studies (and selected for optimal monitoring of a 28-day microbicide-releasing ring in women), a battery life of 450 days is anticipated.

## Discussion

In order to be effective, HIV microbicide products must demonstrate clinical efficacy *and* be used correctly and consistently. Six Phase III studies of vaginal HIV microbicides have been completed to date. All of the products tested were aqueous-based vaginal gels administered by an applicator, and self-reported questionnaires were the primary method used to record adherence in each of these studies. Recognizing the significant drawbacks associated with self-reporting [3,13,14], the most recently completed Phase III studies also included applicator return counts and, in the Carraguard gel trial, an applicator dye test in efforts to more objectively measure adherence [13].

Limited efficacy and related adherence issues with coitally or daily-administered microbicide gel products have directed the microbicide field to focus its attention on vaginal rings, designed to be worn continuously over many weeks or months while providing sustained or controlled release of one or more antiretroviral drugs [22–26]. In contrast to other HIV prevention technologies being tested, vaginal rings require minimal action on the part of the user to achieve product adherence. However, only two studies evaluating vaginal ring adherence in the context of potential use as an HIV prevention method has been reported to date [27,28], and adherence data for other clinical indications is sparse [29–32]. A matrix-type, silicone elastomer vaginal ring containing 25 mg dapivirine, a potent non-nucleoside reverse transcriptase inhibitor, is presently been tested in two Phase III studies in Africa. Although no adherence data is currently available for the dapivirine ring, a recent Phase I trial of a related combination ring product containing 25 mg dapivirine and 100 mg maraviroc (a CCR5 entry inhibitor) reported more than 90% adherence based on self-report, which was confirmed by residual drug levels in used rings [28]. However, it has not yet been demonstrated unequivocally that quantification of residual drug content in used vaginal rings correlates with extent of adherence. Given the inherent variability in drug release from vaginal rings in women as inferred from measured residual drug content values and drug pharmacokinetics [1,28], and the long duration of use of vaginal rings, the single data point obtained using residual drug content analysis may not be sufficiently precise to reliably differentiate between full adherence and, for example, 90% adherence. Additional methods for monitoring ring adherence are therefore needed. The results reported in this study clearly demonstrate that a silicone elastomer vaginal ring containing a DST nano-T temperature logger device is sufficiently responsive to temperature changes upon insertion and removal to offer potential in the quantitative monitoring of product adherence. One of the critical advantages of this approach over drug detection assay is the possibility to distinguish episodes of removal and correlate these with the user experience. For example, if women are routinely removing a ring device during menstruation or coitus, the temperature data should allow the clinical team to identify this repetitive behaviour and develop strategies to improve user adherence for all trial participants.

A single DST nano-T temperature logger currently costs approximately €250 (US$340 at July 2014 exchange rate). There is scope in the longer term for substantially reducing the unit cost of the temperature loggers by scaling production volumes, automating manufacture and decreasing accuracy specifications. However, given the current high cost of the logger relative to the anticipated low-dollar cost of a microbicide-releasing vaginal ring, an inexpensive, temperature-recording, microbicide-releasing vaginal ring intended for widespread use in developing countries is most likely unattainable, at least in the short term. Instead, we propose that a priority objective should be to assess the potential of vaginal rings incorporating temperature loggers to quantitatively measure product adherence in late-stage clinical trials. At least two different types of vaginal ring design may be envisaged for incorporation of a temperature logger device. In the first, referred to as the ‘closed-body’ design, the temperature logger is inserted into a custom mold before being completely encapsulated by the drug-loaded polymer in a single injection-molding step. With this design, the logger could not be easily removed without destroying the integrity of the ring device. Also, for rings manufactured from opaque materials such as filler-loaded or drug-loaded silicone elastomer, the embedded temperature logger would not be visible. Given the typically larger dimensions of silicone elastomer vaginal rings compared with those manufactured from thermoplastics [26], it would be possible to develop a ring design such that the logger was practically indiscernible within the ring body, permitting clinical trials to be designed where women are randomly allocated rings with and without temperature loggers. By comparison, an ‘open-body’ vaginal ring design (Fig 1B), comprising a ring body with a specially-shaped cavity for insertion and retention of the logger, would allow the user or health worker to remove and/or replace the logger. This feature would be potentially useful where the battery life of the logger greatly exceeded the intended duration of ring use, such that the logger could be readily transferred to a new ring (after removal of the logger from the first ring device, data download and memory reset). Both these approaches would be particularly suitable for the manufacture of microbicide-releasing, silicone elastomer, matrix-type vaginal rings similar to the dapivirine-releasing ring [23,26].

The DST nano-T logger contains a silver oxide battery designed to operate optimally close to room temperature. Therefore, using the logger at body temperature will not adversely affect the battery life. A pertinent issue is the battery performance under conditions when the logger is not scheduled to record temperature, such as during storage of a ring device. There are two specific aspects to consider here: (i) the self discharge rate and the sleep current of the logger, and (ii) calibration of the logger. Based on the data presented in Fig 8, the maximum battery life is calculated at 505 days, during which the sleep current of the logger drains the battery. The sleep current increases significantly below 0°C. The self-discharge rate of the battery is typically 5% per year. These values indicate that rings could be manufactured pre-fitted with loggers and comfortably achieve a shelf life of 2–3 years. However, the calibration specifications of the logger is currently only guaranteed for 0.2°C accuracy within the first year of operation, which may reduce the shelf life.

The DST nano-T temperature loggers used in this study had a memory capacity of 5,248 measurements. Recently, Star-Oddi released a second-generation version of the logger capable of recording 43,476 temperature measurements and a claimed battery life of 14 months with a 10 min sampling interval at room temperature. Based on an 8 min sampling interval, this new logger could be used to capture temperature data over 347,808 min (241 days), with a battery life of 451 days. Extending the sampling interval to just over 12.1 min would allow one year of temperature data to be recorded; one year is the longest continuous use period that has been or is being considered for single application of a vaginal ring [33], although vaginally-applied devices that provide microbicide release for multiple years have been reported previously [34,35].

Clinical trials for microbicide loaded vaginal rings often occur in climates that can have maximum daily temperatures significantly greater than those used as part of this study. The technical capabilities of the temperature logger remain unaffected up to 60°C, which is in excess of the highest ever temperature recorded on earth.

Internal vaginal temperature as measured by a radiotelemetric vaginal ring device has been reported to decrease relative to baseline during both non-penetrative sexual arousal and vaginal intercourse, and has been attributed to vaginal wall edema and/or possibly by change of position of the uterus [36]. By comparison, external vaginal temperature measured using either a thermistor attached to a labium minus by means of a padded clip or via thermography directed at a labium majus showed significant increases in temperature during the watching of sexually explicit video material [37,38]. Therefore, in addition to monitoring adherence, a temperature-recording vaginal ring could also be used to monitor the frequency and duration of sexual arousal/intercourse during those times when the ring remains in place. A single vaginal ring device offering extended delivery of one or more anti-HIV microbicides while simultaneously gathering temperature data useful for monitoring both adherence and the frequency of sexual activity has significant clinical potential. Further developments in miniaturisation in other sensor types such as strain gauges and pH meters offer the potential to further enhance data acquisition from ring based devices in the monitoring of sexual encounters of trial participants.

A further potential use of vaginal temperature data is in the identification of a women’s menstrual cycle. This data could provide an extra layer of clinically significant information to help identify if adherence failures are linked to particular time periods within a women’s menstrual cycle.

## Conclusions

The incorporation of a miniature temperature-recording device into a silicone elastomer vaginal ring device can accurately and continuously monitor changes in body temperature, both those small changes associated with the normal diurnal pattern and the larger changes associated with ring removal and re-insertion. Given the concerns over low user adherence and inconsistent product use patterns in clinical studies with vaginally administered HIV microbicides (including vaginal rings) [3], the data presented suggests that a vaginal ring fitted with a temperature logging device could provide a simple biological measure of adherence.

## Supporting Information

S1 Dataset Raw data for temperature logging experiments. (ZIP)

S1 Images Original image files for Fig 1. (ZIP)
