# Supplementary figures and images for: A Temperature-Monitoring Vaginal Ring for Measuring Adherence
Source: PLoS One. 2015 May 12;10(5):e0125682. doi: 10.1371/journal.pone.0125682 (PMC4429109; doi:10.1371/journal.pone.0125682)

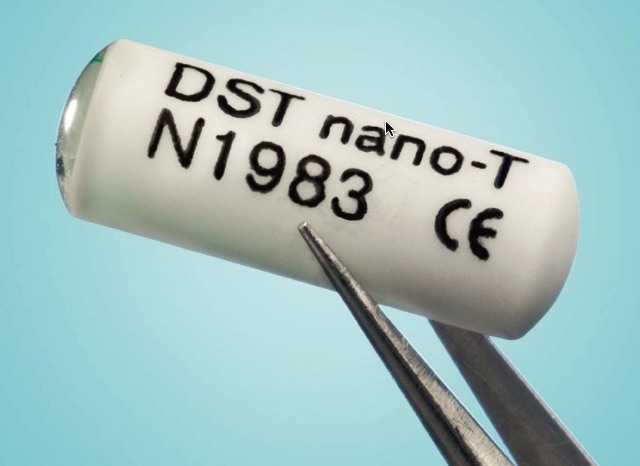

Supplement: S1 Images — (ZIP) [file pone.0125682.s002.zip › DST nano-T Starr Oddi.jpg]

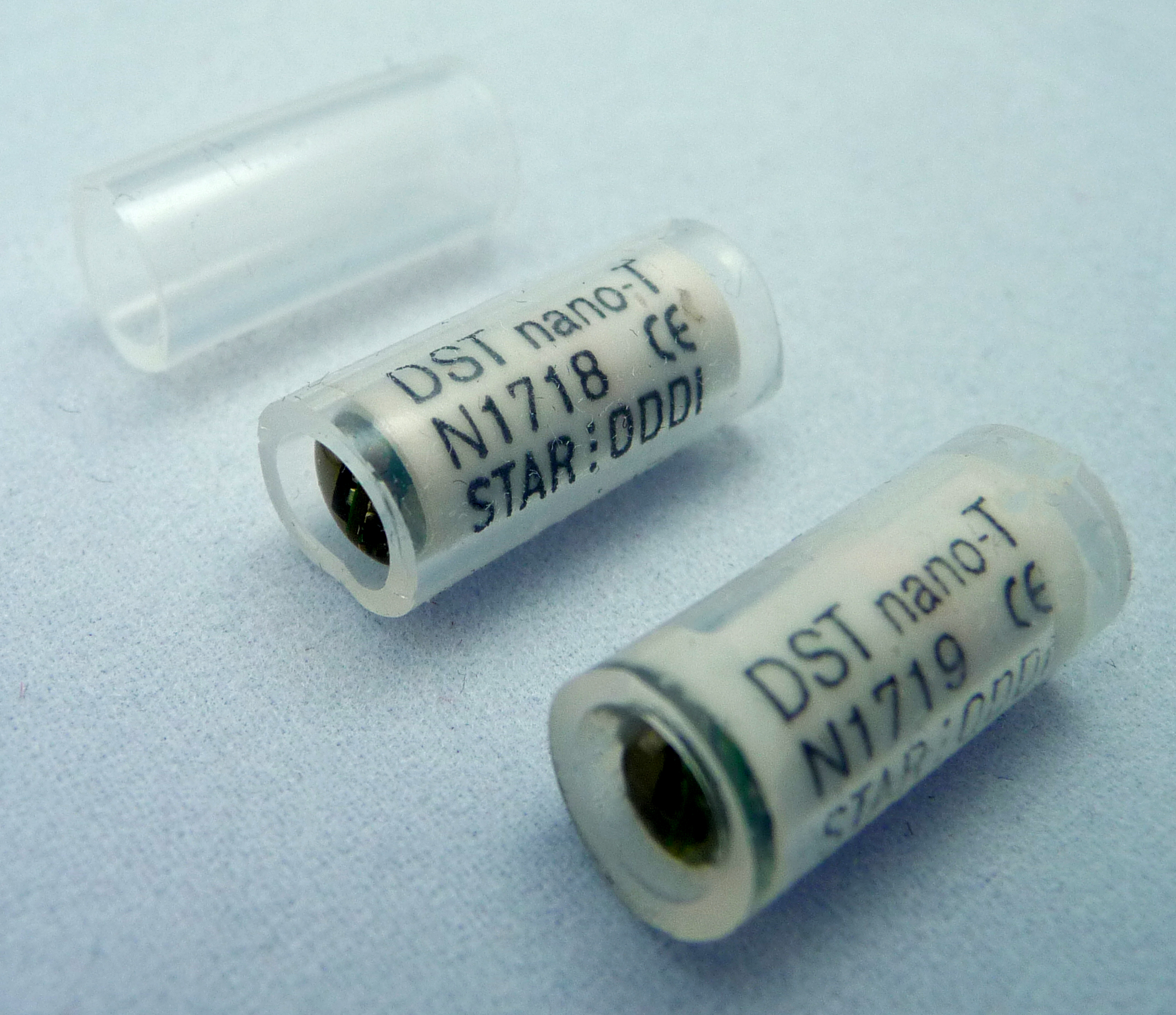

Supplement: S1 Images — (ZIP) [file pone.0125682.s002.zip › P1040333 copy.JPG]

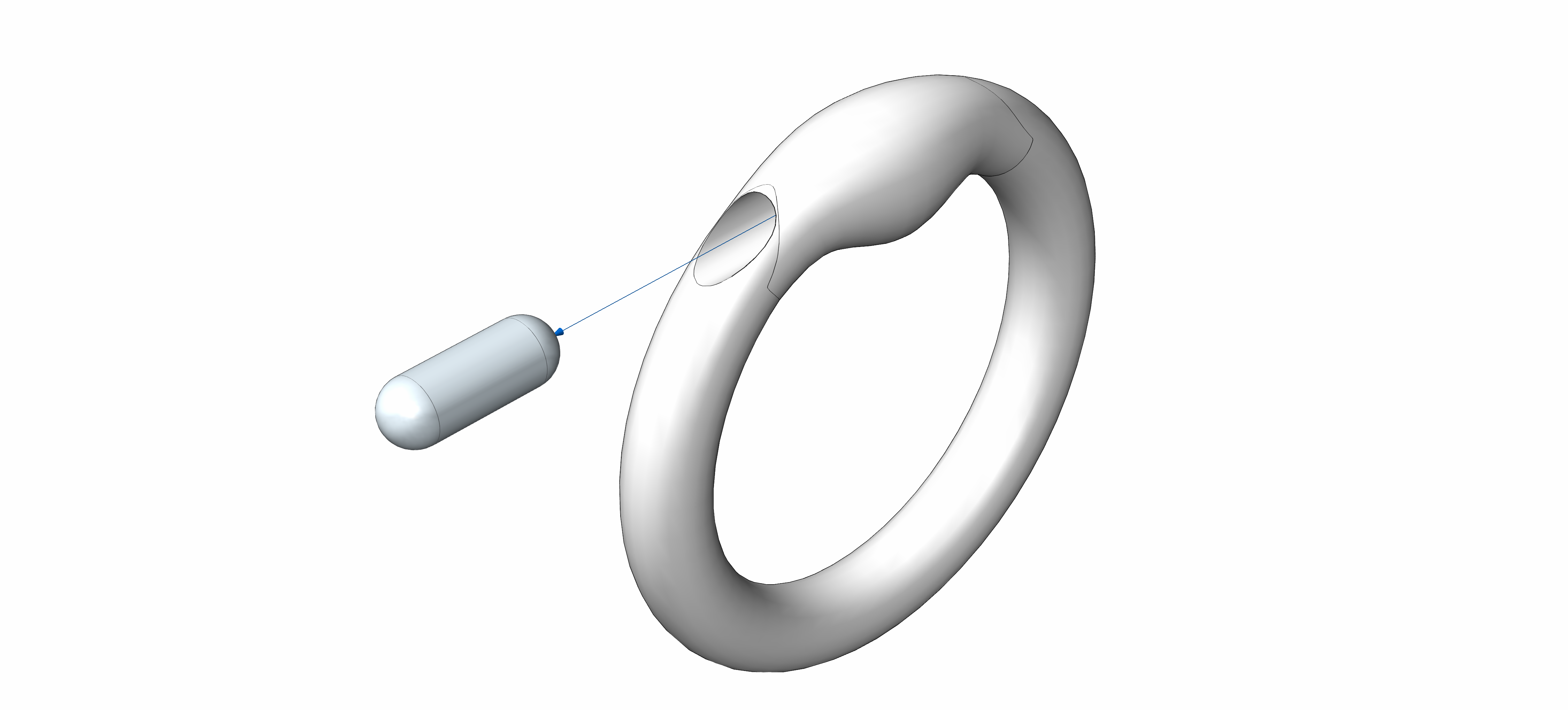

Supplement: S1 Images — (ZIP) [file pone.0125682.s002.zip › Temp logger ring plus logger.tif]
